# Preparing a Liposome-Aided Drug Delivery System: The Entrapment and Release Profiles of Doxorubicin and 9-(*N*-Piperazinyl)-5-methyl-12(*H*)-quino [3,4-b][1,4]benzothiazinium Chloride with Human Serum Albumin

**DOI:** 10.3390/pharmaceutics17020202

**Published:** 2025-02-06

**Authors:** Danuta Pentak, Violetta Kozik, Andrzej Zieba, Marlena Paździor-Heiske, Aleksandra Szymczyk, Josef Jampilek, Andrzej Bak

**Affiliations:** 1 Faculty of Chemistry and Pharmacy, University of Opole, Oleska 48, 45-052 Opole, Poland; danuta.pentak@uni.opole.pl; 2 Institute of Chemistry, University of Silesia, Szkolna 9, 40-006 Katowice, Poland; violetta.kozik@us.edu.pl (V.K.); pazdzior.marlena@gmail.com (M.P.-H.);; 3 Department of Organic Chemistry, Faculty of Pharmaceutical Sciences in Sosnowiec, Medical University of Silesia in Katowice, Jagiellońska 4, 41-200 Sosnowiec, Poland; zieba@sum.edu.pl

**Keywords:** liposomes, drug release profile, human serum albumin, controlled drug delivery systems, ANOVA

## Abstract

**Background/Objectives:** The principal aim of this work was to prepare a liposomal drug delivery system based on the commercial drug doxorubicin (DOX) and a budding agent with promising anticancer activity, 9-(*N*-piperazinyl)-5-methyl-12(*H*)-quino [3,4-b][1,4]benzothiazinium chloride (9-PBThACl). **Methods**: A spectrophotometric methodology was used to meticulously investigate the drug entrapment and release characteristics of the new liposomal complexes (L) based on dipalmitoylphosphatidylcholine (DPPC) with human serum albumin (HSA) and its defeated analog (dHSA). **Results**: The impact of the operational parameters (temperature and pH) on the liposome/drug(s)/(d)HSA, namely [L_DPPC/9-PBThACl/DOX_ ]:(d)HSA] systems, as well as the polarity of the phospholipid bilayer, was examined. In order to compare the experimental findings, mathematical models were employed to specify the analytical factors controlling the process of drug release/potential drug release from liposomes. The observed variations in the drug encapsulation and release profiles were due to the combination of liposomal conjugates with human plasma protein. **Conclusions**: It was proven that changes in the environmental pH directly affect the percentage of drug entrapment in liposomes and the medicine release efficiency. Moreover, the grouping tendency of the liposomal combinations was investigated using a principal component analysis (PCA) and a hierarchical clustering analysis (HCA). Finally, an analysis of variance (ANOVA) confirmed the statistical impact of pH buffering and changing temperature factors on the drug release characteristics of liposomal conjugates.

## 1. Introduction

Human serum albumin (HSA) is a widely distributed plasma protein in nature that has attracted much attention in the development of drug delivery systems [1,2,3,4]. Due to the versatile properties of HSA, HSA-based nanoparticles are regarded as attractive therapeutic agents, characterized by desirable pharmaceutical, pharmacokinetic, and pharmacodynamic profiles with enhanced efficacy, reduced toxicity, or an extended circulation half-life [5,6,7]. As a matter of fact, the protein’s ability to bind ligands (e.g., drug molecules) and act as a carrier protein (or drug vehicle) for many exo- and endogenous compounds has been extensively scrutinized so far [8,9,10]. Hence, a significant amount of data, including the locations of binding pockets and potential guest–host (ligand-receptor) interactions, have been periodically obtained [11,12].

**Figure 1:**
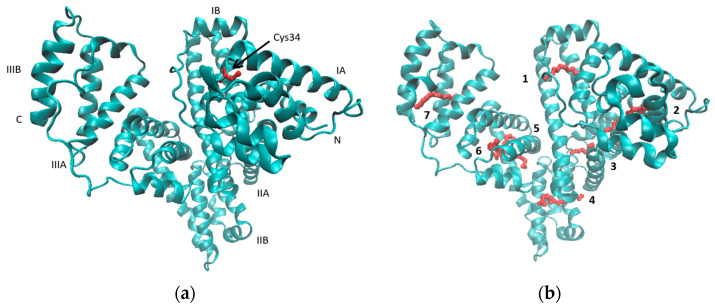
Crystal structures of HSA with the position of Cys34 indicated in red (**a**). The subdivision of the protein into domains (I–III) and subdomains (A, B). PDB id code: 1bm0. The locations of the seven (1–7) binding sites common to fatty acid anions using palmitate are presented in red (**b**). PDB id code: 1e7h.

HSA is a multifunctional protein synthesized exclusively by liver hepatocytes and continuously secreted into the blood circulation system with a fairly long half-life of 19 days. It is the most abundant protein, accounting for 60–65% of the total plasma protein; the total amount of HSA in the body is about 360 g, which is approximately one-third of the bloodstream (the concentration of 35–50 g/L). HSA is observed to be synthesized as a simple, monomeric protein without prosthetic groups and covalently bound lipids or carbohydrates. In fact, the geometrical 3D structure of a single polypeptide chain and its recombinant version (rHSA) has been crystallographically specified with a satisfactory resolution of 2.5 Å [13,14]. The polypeptide chain forms a heart-shaped protein with approximate dimensions of 80 × 80 × 80 Å and a thickness of 30 Å, where 67% composes a regular α helix structure. HSA comprises three homologous domains (I–III), each consisting of two subdomains, A and B, with six and four α helices connected by flexible loops, respectively. All but 1 (Cys34) of the 35 cysteine residues are involved in forming 17 stabilizing disulfide S-S bonds, as illustrated in Figure 1.

Small-angle X-ray scattering studies of HSA in a solution revealed the general agreement with the crystal structure [15]. Furthermore, a combined phosphorescence depolarization hydrodynamic modeling study proved that the overall conformation of HSA in a neutral solution aligns with the one observed in crystal structures [16]. 

Not surprisingly, the application of many drugs, for instance, chemotherapeutic or gene therapy agents, is hampered by the drug’s poor solubility in aqueous media, marked toxicity, and/or rapid degradation in vivo. In order to address the issues of drug distribution from the place of administration to the site of interaction, nanoparticles might be used as carriers (named ‘Trojan horses’) of active substances. It seems that artificially structured vesicles composed of a phospholipid bilayer (liposomes) are frequently used as nanoscale carriers to encapsulate medicines in controlled drug delivery systems. In this context, albumin appears to be a promising entrapment medium due to its stability, biodegradability, non-toxicity, non-immunogenicity, and ability to bind various drug molecules [17].

In general, HSA serves as a transport protein exerting a mutually noticeable impact on the drug’s ADMET-related properties (absorption, distribution, metabolism, excretion, and toxicity). Interestingly, the binding of albumin may also affect ligands more individually, resulting in the increased solubility of the compound in plasma or the protection of the ligand against oxidizing agents. Despite the universal ligand-binding potential, HSA mainly binds organic anions and inorganic cations. In fact, the unique ability of the HSA protein to bind the drug molecule is mainly induced by the favorable combination of hydrophobic pockets, side chain charge distributions, and side chain flexibility, which is largely due to relatively long interdomains and intradomain polypeptide linkers and flexible loops [18]. The albumin-bound paclitaxel (Abraxane^®^) was the first commercially available protein-based nanoparticle (approved by the FDA in 2005) successfully applied in oncology. Thence, a range of albumin-based carriers containing other active compounds, such as doxorubicin [19,20], cisplatin [21,22], docetaxel [23,24], rapamycin [25], vincristine [26], mitoxantrone [27], ferulate [28], and iron oxide [29,30], were produced. It is known that the primary purpose of making such particles is their potential application to medicalize various forms of cancer and vascular diseases, as well as for uses in imaging and chelation therapy. In practice, trapped drugs are being extensively scrutinized at different phases of clinical trials. It was noticed that using nanoparticles improves the drug’s biodistribution (pharmacokinetics and pharmacodynamics) pattern, including bioavailability, the release time of the active substance, and the extended duration of pharmacological action. Synthetically, nanoparticles are frequently prepared for therapeutic purposes in the form of liposomes, fullerenes, nanotubes, and dendrimers; however, in medicine, the most commonly analyzed are liposomes → spherically shaped media made of a single- or multi-layer lipid shell enclosed inside an aqueous space (liposomal core). Naturally, the liposomal shell is constructed analogously to biological membranes that facilitate the transport of both lipophilic drugs (located in the free spaces of the lipid layer) and hydrophilic substances (placed in the water solution). Unfortunately, the observed constraints in using liposomes are their rapid uptake and degradation by liver macrophages, which shorten the duration of action of the drug transported in the body. Luckily, the duration of liposomal interaction within the human body might be noticeably extended by generating a [liposome/drug]–albumin complex [31,32]. A unique feature that makes albumin so attractive as an effective drug carrier is its ability to bind with receptors overexpressed by tumors. The main metabolic path on which albumin depends during internalization inside the tumor is the transcytosis endothelial path, where the protein intercedes. Albumin is characterized by a fairly high affinity to the gp60 receptor (albondin) with a mass of 60 kDa. Additionally, the glycoprotein surface receptors gp18 and gp30 with masses of 18 and 30 kDa are targeted by albumin as well. It should be highlighted that gp60 allows albumin to participate in the specific targeting of cancerous cells because it mediates the transportation of medicines in albumin-based drug delivery systems via biological, epithelial, and endothelial barriers, undergoing overexpression in cancer cells [33]. A detailed study of albumin’s interactions with liposomal structures showed that proteins partially penetrate and deform the lipid bilayer [34]. To some extent, HSA penetrates liposomal bubbles partially being adsorbed on its surface. 

The marketed drug doxorubicin (DOX) forms complexes with DNA by intercalating between pairs of bases and inhibits topoisomerase II activity via stabilizing the DNA–topoisomerase II complex due to the partial prevention of the ligation reactions that are catalyzed by the topoisomerase II enzyme [35,36]. As a representative anthracycline antibiotic, doxorubicin exerts a cardiotoxic effect and exhibits antimitotic and cytotoxic activity. Conducted clinical studies revealed a reduced number of cardiac events in patients treated with liposomal DOX compared to those medicated using the conventional DOX; for instance, a statistically significant lower incidence of heart failure was reported [37,38]. On the other hand, the liposome-based DOX exhibited a similar activity against doxorubicin-resistant cell lines. Moreover, the liposome-encapsulated DOX showed reduced drug distribution to the heart, gastric mucosa, and intestines while maintaining antitumor efficacy. Drug encapsulation seems to protect the patient from the adverse effects of the chemotherapeutics used.

Neuroleptic phenothiazines containing alkylaminoalkyl substituents at the thiazine nitrogen atom exhibit antitumor activity; therefore, we proposed a new method of tetracyclic phenothiazine derivative synthesis [39,40]. Briefly, the structure of the synthesized compounds was modified by introducing different pharmacophoric groups in the 9-, 10-, and 11-positions of the tetracyclic quinobenzothiazine system, revealing the dependence of antitumor activity not only on the nature of the pharmacophoric groups but also on their position in the tetracyclic quinobenzothiazine system [41]. In fact, the most active derivatives showed anticancer activity comparable to drugs currently used in chemotherapy (e.g., doxorubicin and cisplatin); therefore, the most probable mechanism of action is the intercalation of the DNA helix of cancer cells [42]. The most potent molecules contain the substituents (e.g., amino groups) in the 9- or 10-position of the tetracyclic system that form hydrogen bonds (HBs) with purine and pyrimidine bases of the DNA helix stabilizing the compound–DNA complex. Hence, 9-(*N*-piperazinyl)-5-methyl-12(*H*)-quino [3,4-*b*][1,4]benzothiazinium chloride (9-PBThACl), containing a piperazyl substituent in the 9-position, was examined as an antiproliferative agent with a promising IC_50_ value (compared to cisplatin as a reference drug molecule).

The choice of the liposome administration route depends on the type of cancer, the properties of the drug, and the intended therapeutic effect. In fact, some anticancer or anti-inflammatory drugs are designed to work more effectively in an acidic pH environment. The proposed carriers indicate the difference in drug release in response to pH changes. The effect of pH on the release and action of the drug is a key element of pharmacokinetics; therefore, the selected temperatures were intended to replicate physiological conditions, with 37 °C representing the normal state and 41 °C representing an inflammatory state.

The molecular entrapment and the release characteristics of liposomal delivery systems, including the promising anticancer agent 9-(*N*-piperazinyl)-5-methyl-12(*H*)-quino [3,4-*b*][1,4]benzothiazinium chloride (9-PBThACl) and the marketed drug doxorubicin (DOX), were investigated in the presence of serum albumin molecules HSA and dHSA (deprived of endogenous fatty acids). In practice, new liposomal complexes 1–12 with human serum albumin ([L_DPPC/9-PBThACl/DOX_ ]:HSA vs. [L_DPPC/9-PBThACl/DOX_]:dHSA) were experimentally and theoretically examined in the changing operational conditions of temperature and pH using the spectrophotometric methodology and modeling procedures as well.

## 2. Materials and Methods

### 2.1. Materials

1,2-Dipalmitoyl-sn-glycero-3-phosphocholine (DPPC, ≥99%), dichloromethane, chloroform, potassium phosphate dibasic (≥98%), sodium phosphate monobasic monohydrate (≥98%), and doxorubicin hydrochloride (98.0–102.0% (HPLC)) were purchased from Sigma Aldrich, Schnelldorf, Germany; albumin from fatty acid-free and globulin-free human serum lyophilized powder (≥99%) was from Sigma Aldrich; and albumin from human serum fraction V, high purity, was from EMD Millipore, Germany. 9-(N-piperazinyl)-5-methyl-12(H)-quino [3,4-b][1,4]benzothiazinium chloride (9-PBThACl) was synthesized at the Department of Organic Chemistry, Medical University of Silesia in Katowice, Poland.

### 2.2. Liposome Preparation

The modified reverse-phase evaporation method (mREV) was used to produce the liposome conjugates. The mREV methodology consists of the continuous mixing of specific volumes of appropriate phospholipid solutions in excess of organic solvents with a water phase following the procedure described in detail by Papahadjopoulos [43]. The proposed adjustment of the above method involves substituting the ultrasonic dispersion of the aqueous phase in the organic phase with mechanical dispersion, which enables the comprehensive conversion of phospholipids to liposomes. The DPPC:drug molar ratio of 30:1 and the DPPC:drug:drug molar ratio of 30:1:1 were applied in order to prepare liposomes (L_DPPC_; L_DPPC/9-PBThAC_; L_DPPC/9-PBThACl/DOX_) using the mREV approach. Lipid dispersion at a final lipid concentration of ca. 2.64 × 10^−2^ M was used. In practice, a total of 0.34 mL of 9-PBThACl (the synthesis of 9-PBThACl was described previously in [44]) and doxorubicin at a concentration of 5 × 10^−3^ M were added to the mixture. Briefly, equal amounts (0.34 mL) of 9-PBThACl and DOX at a concentration of 5 × 10^−3^ were mixed with lipid dispersion. An amount of 2 mL of PBS buffer with the appropriate pH value and 4 mL of an organic solution prepared from dichloromethane and chloroform were applied. The average time required for liposome preparation did not exceed 12 min. The method used is fast, does not generate high costs, and allows for the complete transformation of phospholipids into liposomes.

### 2.3. Solutions and Sample Preparation

Phosphate-buffered saline (PBS) at pH = 5.50, 6.00, 6.50, and 7.40 was employed in the UV/Vis measurements. A stock solution of 9-PBThACl/DOX in H_2_O (5.0 × 10^−3^ M) was prepared for the examination of the human serum albumin–drug interaction. Stock solutions of human serum albumin (HSA and dHSA) at the concentration of 1.0 × 10^−3^ M were used at the specific pH of the environment. All mixtures used in the reactions were prepared in triplicate, fulfilling the statistical analysis requirements.

### 2.4. UV/Vis Measurements

The absorption spectra of pure 9-PBThACl, doxorubicin, the liposomal form of 9-PBThACl, and doxorubicin with HSA and dHSA were recorded using a Lambda Bio 40 spectrometer (Perkin Elmer, Waltham, MA, USA) equipped with a PTP-1 Peltier System (Perkin Elmer, Waltham, MA, USA) automatic temperature controller. The temperature was controlled in the range of 37 ± 0.1 °C and 41 ± 0.1 °C. All spectra were recorded after the equilibration of the samples with the automatic temperature controller. The spectral examination was performed using UV WinLab Perkin Elmer Software (V6.0.4).

### 2.5. Drug Release Versus Mathematical Modeling

The specification of 9-PBThACl, DOX release from liposomes [L_DPPC/9-PBThACl/DOX_]: HSA, and [L_DPPC/9-PBThACl/DOX_]:dHSA was carried out at 37 °C and 41 °C in a period of 5 h. Spectrophotometry was employed to examine the degree of drug release from liposomes. During the first 60 min of experiments, the measurements were recorded in 5 min intervals. Subsequently, the surveys were recorded every 15 min. Drug release (R%) was calculated according to Equations (1) and (2) [45].

(1) $$R\left(\%\right)=\frac{\left([x{]}_{f\;}-[x{]}_{fo}\right)}{\left([x{]}_{t}\times EE\%\right)}\times 100$$

where

$R\left(\%\right)$—encapsulated drug percentage of release;

$[x{]}_{f\;}$—concentration of released drug molecule;

$[x{]}_{fo}$—initial concentration of unencapsulated drug;

EE%—percentage of drug encapsulation.

(2) $${\left[x\right]}_{t}=\frac{{\left[x\right]}_{to}}{\beta }$$

where

${\left[x\right]}_{to}$—concentration of total drug in original liposomes;

β—dilution.

In order to theoretically estimate the release process and the degree of drug leakage from the liposomes, a set of mathematical models [46,47,48] was implemented as follows:

(3) $$\mathrm{First}–\mathrm{order}:\;X=1-e^{-k(t-\alpha )}$$

(4) $$\mathrm{Bhaskas}:\;X={1-e}^{{-k(t-\alpha )}^{0.65}}$$

(5) $$\mathrm{Higuchi}:\;X={k(t-\alpha )}^{0.5}$$

(6) $$\mathrm{Ritger}–\mathrm{Peppas}:\;X={k(t-\alpha )}^{n}$$

(7) $$\mathrm{Korsmeyer}–\mathrm{Peppas}:\;X={kt}^{n}$$

(8) $$\mathrm{An}\;\mathrm{extension}\;\mathrm{of}\;\mathrm{the}\;\mathrm{classical}\;\mathrm{Freundlich}\;\mathrm{model}:\;X={a+kt}^{n}$$

where

*X*—release percentage R [%] based on fitting of empirical data to mathematical equations;

*t*—release time;

*k*—kinetic constant;

*α*—modified parameter;

*a*—fit factor;

*n*—exponent factor depicting various mechanisms of drug leakage.

The numerical value of *n* < 0.45 corresponds to Fick’s diffusion, and the square root of the time value is then proportional to the number of fractions released from the carrier. A process other than Fick’s diffusion is used in the condition that 0.45 < *n* < 0.89, and drug release appears as a consequence of both the diffusion and controlled mechanisms [49]. The residual sum of squares (*SUM*) and *R^2^_adj_* parameters were estimated according to Equations (3)–(8).

### 2.6. Encapsulation Efficiency

Following the preparation of the liposomal formulation containing 9-PBThACl and DOX, the samples were subjected to dialysis to separate the encapsulated molecules from their free forms. A total of 12mL of the liposomal formulation was placed in cellulose ester dialysis tubing (Spectra/Por^®^, MWCO 8000–10,000 Da, Spectrum, Quebec, QC, Canada). The dialysis bag was immersed in 50 mL of buffer solution and maintained at 4 °C. Dialysis was continued until no characteristic peaks of the compounds were detected in the UV/Vis spectrum at wavelengths of 488 nm and 233 nm, indicating the removal of unencapsulated 9-PBThACl and DOX. The concentrations of encapsulated 9-PBThACl and DOX within the liposomes were determined spectrophotometrically by comparing absorbance values to a standard calibration curve. The encapsulation efficiency (EE%) of 9-PBThACl and doxorubicin in the liposomal formulation was calculated using the following equation:

(9) $$EE\%=\frac{{c\;}_{total}-c_{free}}{c_{total}}\times 100$$

where

*c_total_*—total amount of drug;

*c_free_*—free amount of drug in supernatant.

### 2.7. Zeta Potential and Particle Size Measurements

Zeta potential and particle size measurements were performed using electrophoretic light scattering (ELS) on the Zetasizer Advance Malvern Panalytical. The suspended particles begin to move at a characteristic speed called electrophoretic mobility under the influence of an electric field. The zeta parameter can be measured using light scattering with phase analysis (PALS technique), which examines the tiny phase shifts in the scattered light caused by moving particles. The electrophoretic mobility can then be converted to the zeta potential. Dynamic light scattering (DLS) is a widespread method of measuring the size of nanoparticles dispersed in a liquid, where a laser beam illuminates the dispersion of nanoparticles. The laser light is scattered on particles vibrating with Brownian motion and hits the detector. The signal on the detector changes depending on the speed of the particles, which is correlated with their size—small particles move faster, and large ones move slower. The sizes of the particles are determined using the Stokes–Einstein equation. Zetasizer analyzers use the patented non-invasive back scatter (NIBS), where the detector is positioned at an angle of 173°.

### 2.8. Statistical Analysis

All experiments and measurements were conducted in triplicate, and the data were reported in the typical form of the mean with the standard deviation (±SD). The generated findings were analyzed using OriginPro 8.5.0 SR1 software (OriginLab Corporation, Northampton, MA, USA). All PCA, HCA, and ANOVA calculations were performed using MATLAB R2016a (MathWorks, Natick, MA, USA) on a computer running the Windows 11 operating system.

## 3. Results and Discussion

### 3.1. In Vitro Drug Release Evaluation

**Figure 2:**
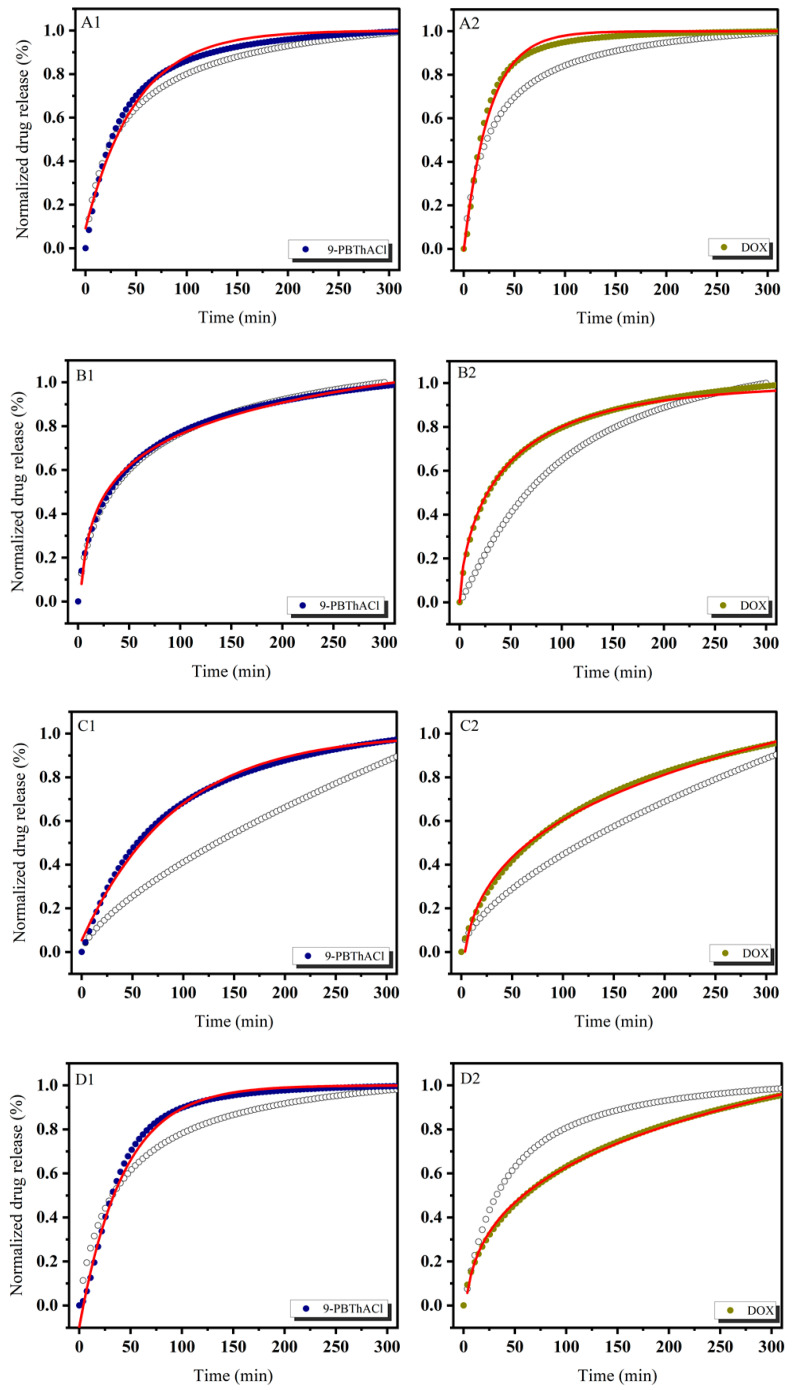
The release kinetics of 9-PBThACl and DOX from the [L_DPPC/9-PBThACl/DOX_]:HSA liposomes. The red lines represent the fitting of the best mathematical models: (A1: pH = 5.5, 9-PBThACl, A2: pH = 5.5, DOX), (B1: pH = 6.0, 9-PBThACl, B2 pH = 6.0, DOX), (C1: pH = 6.5, 9-PBThACl, C2: pH = 6.5, DOX), and (D1: pH = 7.4, 9-PBThACl, D2: pH = 7.4, DOX). Measurement temperature: 37 °C.

**Figure 3:**
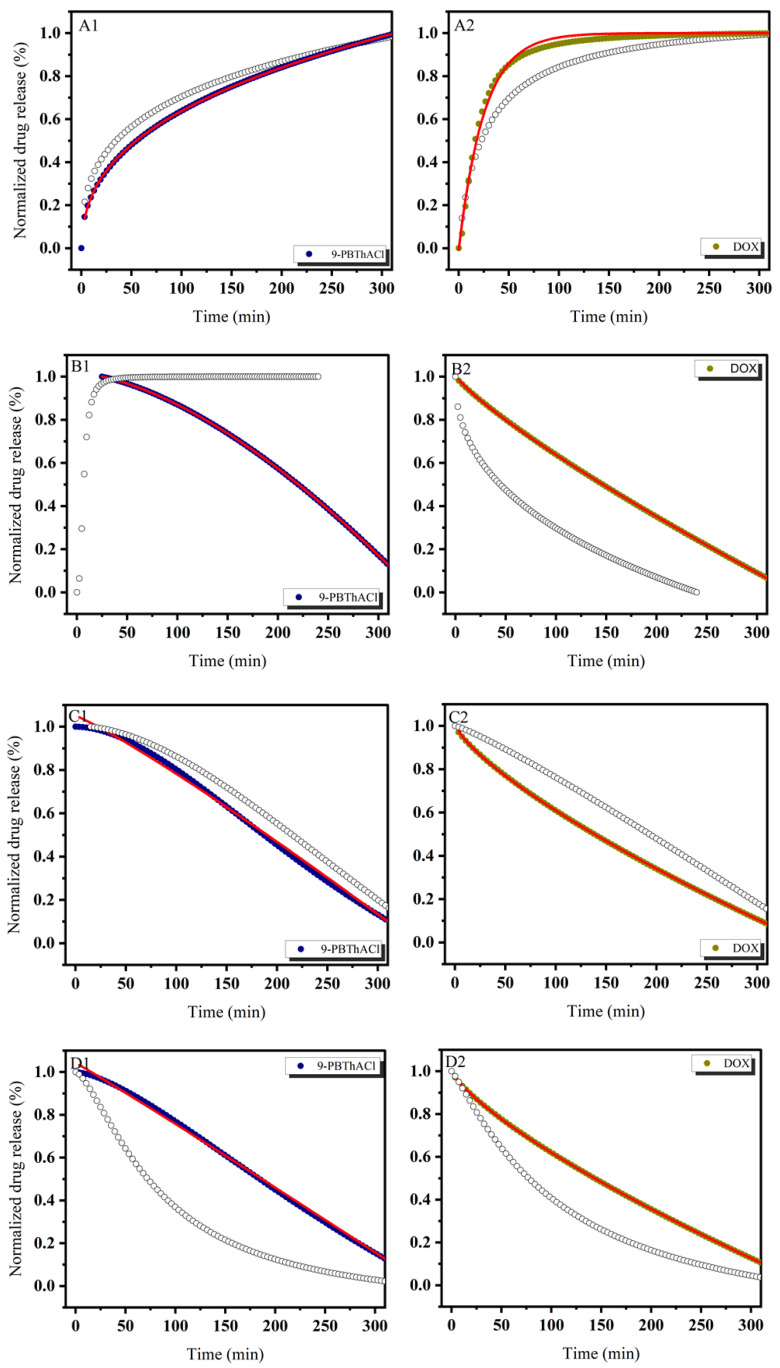
The release kinetics of 9-PBThACl and DOX from the [L_DPPC/9-PBThACl/DOX_]:HSA liposomes. The red lines represent the fitting of the best mathematical models: (A1: pH = 5.5, 9-PBThACl, A2: pH = 5.5, DOX), (B1: pH = 6.0, 9-PBThACl, B2 pH = 6.0, DOX), (C1: pH = 6.5, 9-PBThACl, C2: pH = 6.5, DOX), and (D1: pH = 7.4, 9-PBThACl, D2: pH = 7.4, DOX). Measurement temperature: 41 °C.

**Figure 4:**
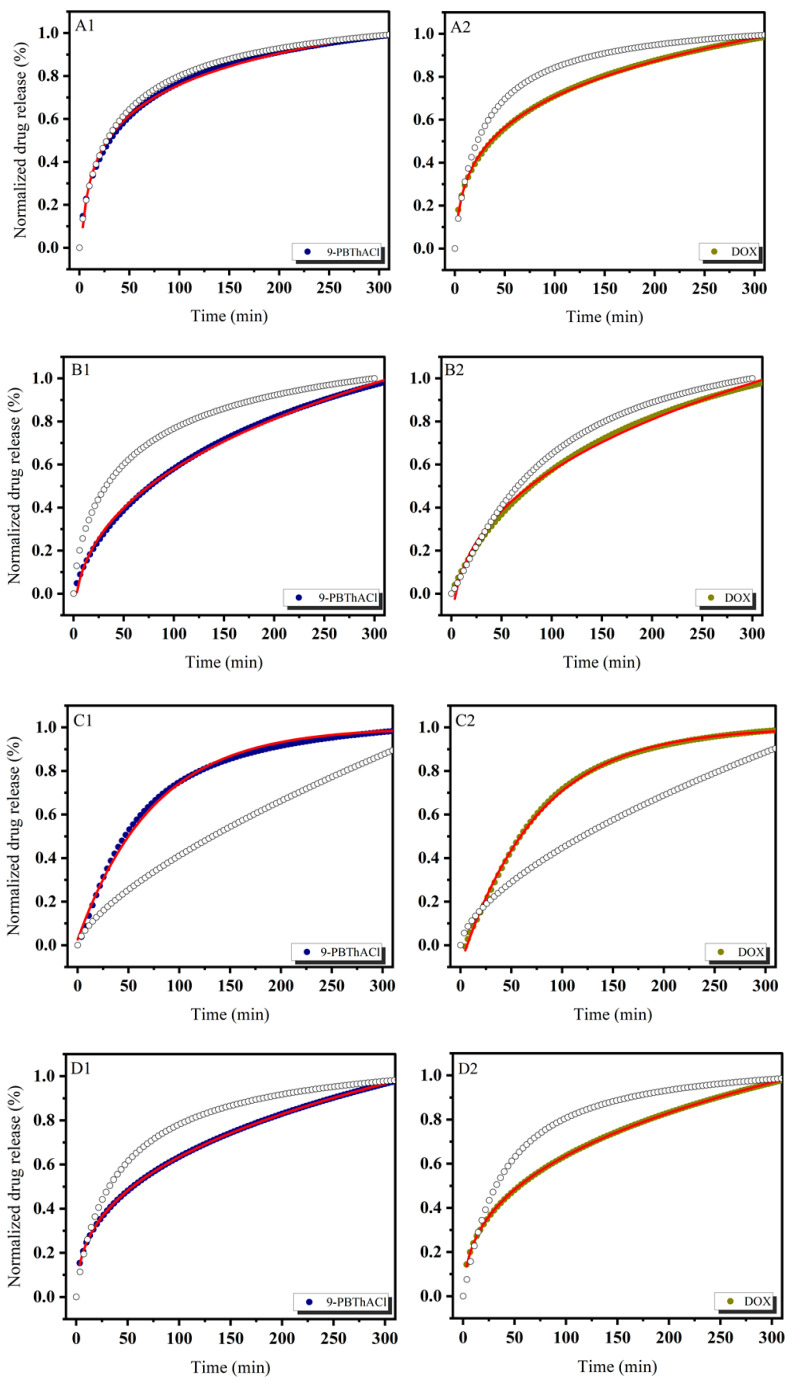
The release kinetics of 9-PBThACl and DOX from the [L_DPPC/9-PBThACl/DOX_]:dHSA liposomes. The red lines represent the fitting of the best mathematical models: (A1: pH = 5.5, 9-PBThACl, A2: pH = 5.5, DOX), (B1: pH = 6.0, 9-PBThACl, B2 pH = 6.0, DOX), (C1: pH = 6.5, 9-PBThACl, C2: pH = 6.5, DOX), and (D1: pH = 7.4, 9-PBThACl, D2: pH = 7.4, DOX). Measurement temperature: 37 °C.

**Figure 5:**
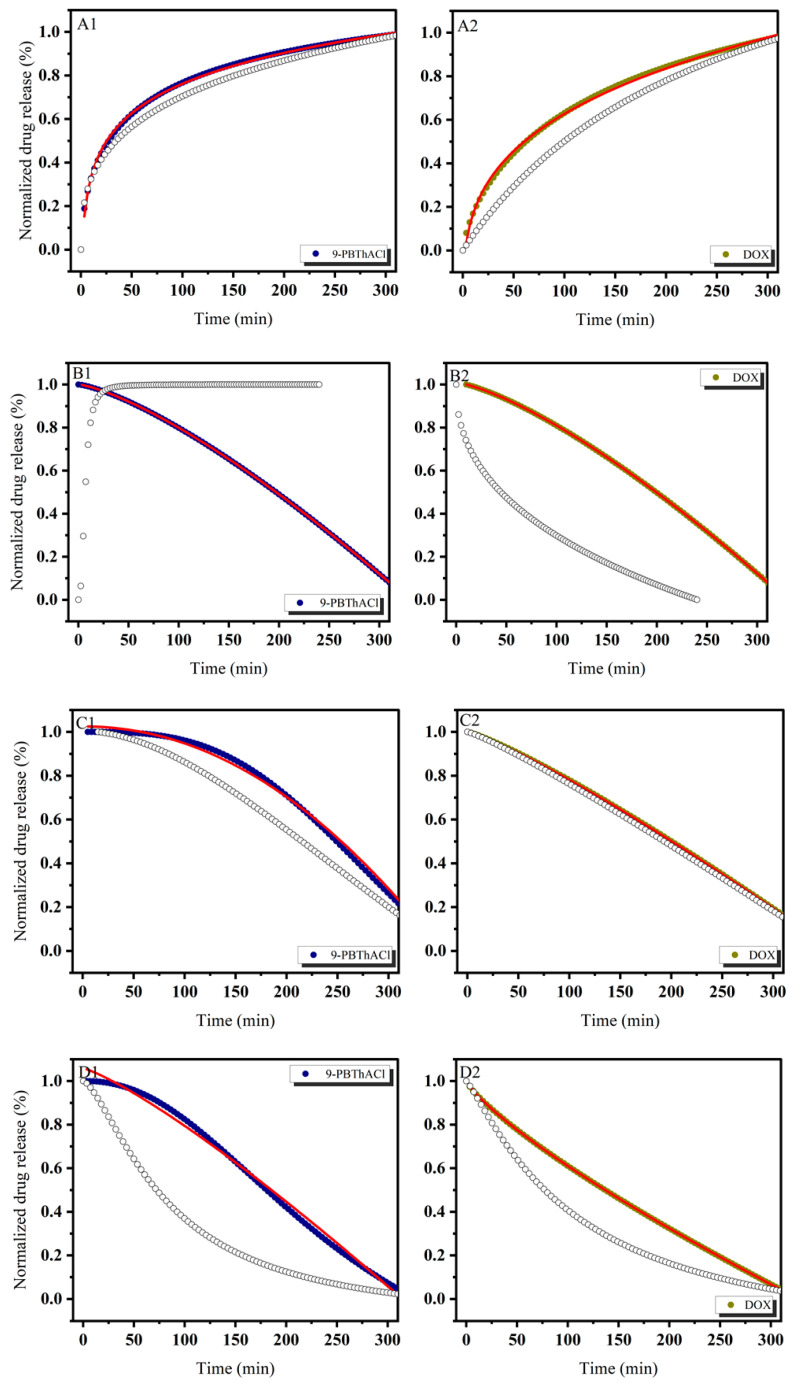
The release kinetics of 9-PBThACl and DOX from the [L_DPPC/9-PBThACl/DOX_]:dHSA liposomes. The red lines represent the fitting of the best mathematical models; (A1: pH = 5.5, 9-PBThACl, A2: pH = 5.5, DOX), (B1: pH = 6.0, 9-PBThACl, B2 pH = 6.0, DOX), (C1: pH = 6.5, 9-PBThACl, C2: pH = 6.5, DOX), and (D1: pH = 7.4, 9-PBThACl, D2: pH = 7.4, DOX). Measurement temperature: 41 °C.

In order to theoretically approach the operational parameters and the degree of drug (DOX) and/or potential drug (9-PBThACl) release from liposomes, six mathematical models were applied, including first-order, Bhaskas, Higuchi, Ritger–Peppas, Korsmeyer–Peppas, and an extension of the classical Freundlich model (see fitting variables reported in Tables S1 and S2). Practically, the release rates of the drug/prospective drug molecules were examined at temperatures of 37 °C as well as 41 °C. Additionally, the procedure of drug entrapment within liposomes was extended by the application of two forms of human serum albumin: HSA and dHSA. The released kinetics of the resulting complexes [L_DPPC/9-PBThACl/DOX_ ]:HSA and [L_DPPC/9-PBThACl/DOX_ ]:dHSA are presented graphically in the functions of time (1–300 min), environment pH (5.5, 6.0, 6.5, 7.4), measurement temperature (37 °C and 41 °C), and liposomal composition conjugated with human serum albumin (HSA and dHSA), as shown in Figure 2, Figure 3, Figure 4 and Figure 5.

The data, illustrated in Figure 2, Figure 3, Figure 4 and Figure 5, confirm the previously presented tendency (see part 1 of our studies [44]), where the direct influence of temperature and environmental pH on the drug release characteristics from liposomes was reported. In general, the applied models, calculated according to Equations (3)–(8), enabled the examination of different aspects of the in vivo drug distribution. A comprehensive review (including the profound examination of various drug release systems with respect to the mechanism of action that restricts the drug from spreading within the body) is beyond the scope of this paper; however, it can be found elsewhere [50]. Overall, the drug release profiles are functionally dependent on diffusion processes (within the material and/or via the membrane), carrier degradation, material bulking, and environmental parameters, e.g., temperature and pH changes.

In practice, the drug release profiles produced at a temperature of 37 °C and the environmental pH values of 5.5, 6.0, 6.5, and 7.4 for the complexes with HSA and dHSA were approximated exponentially, as reported in the Supplementary Materials (see Tables S1 and S2). As a matter of fact, the best fit for HSA was recorded using the first-order model, while for dHSA, an extension of the classical Freundlich model exhibited satisfactory consistency with the experimental data. Conversely, the ultimate match for HSA data was demonstrated at pH = 6.5 by extension of the classical Freundlich model, whereas for dHSA, it was the first-order model at the same pH in the surrounding environment. Similarly, an extension of the classical Freundlich model was used to decrease functions at 41 °C for both HSA and dHSA data, as shown in Tables S1 and S2 in the Supplementary Materials. Not surprisingly, an increase in temperature to the main point of the DPCC phase transition destabilized the tested liposomal conjugates [51]. It appears that an ambient pH can explicitly modify the thermal profiles of drug complexes trapped in liposomes with HSA as well as dHSA proteins. Furthermore, the thermal characteristics of drug release for the [L_DPPC/9-PBThACl/DOX_]:dHSA liposome complex prepared at a temperature of 41 °C and in surrounding buffer with pH = 6.0, 6.5, and 7.4 were described by R^2^_adj_ = 1. In contrast, the recorded drug release pattern varied considerably from the system generated at pH = 5.5. Thus, the pH of the environment determines the specific conformation of phospholipids, which translates into the kinetics of releasing the compound enclosed in liposomes.

### 3.2. Characteristics of Liposomes

The zeta potential (ζ) depends on the surface charge and is essential for the stability of nanoparticles in suspension [52]. It is also a significant factor in the initial adsorption of nanoparticles on the cell membrane. The electrokinetic potential of liposomes is related to the presence of a double electrical layer on the surface of the particles with oppositely charged ions. The zeta potential is revealed by the mutual motion of particles relative to the medium—it is defined as the potential occurring at the surface interface separating the particle (liposome) with adsorbed ions from the rest of the double layer [53]. In the case of high and negative or positive potential, due to electrostatic repulsion, the system is characterized by good stability and is less susceptible to chemical degradation or aggregation. On the other hand, a potential close to 0 indicates low system stability, which adversely affects the properties of liposomes, which in turn fuse to form larger aggregates. Biodistribution and the rate of therapeutic removal by macrophages of the reticuloendothelial system (RES) depend on the sizes of the liposomes [54]. In fact, large liposomes with a diameter (d) of about 1000nm are recognized to be removed faster by macrophages compared to smaller liposomes (d ≈ 100 nm), which show increased accumulation in tumor tissue and a longer circulation time in the blood. Liposomes with a size in the range of 100–1000 nm are responsible for efficient drug delivery to tumors using the effect of increased permeability and retention (EPR).

In order to provide the basic characteristic of the generated liposomal systems, the zeta potential (ζ = −1.8 ± 07 mV) and liposome particle size (67–68 nm) were measured three times at a temperature of 250 °C. A low negative zeta potential result was obtained, which affects liposome aggregation after several weeks.

### 3.3. Similarity-Guided Drug Release Evaluation

**Figure 6:**
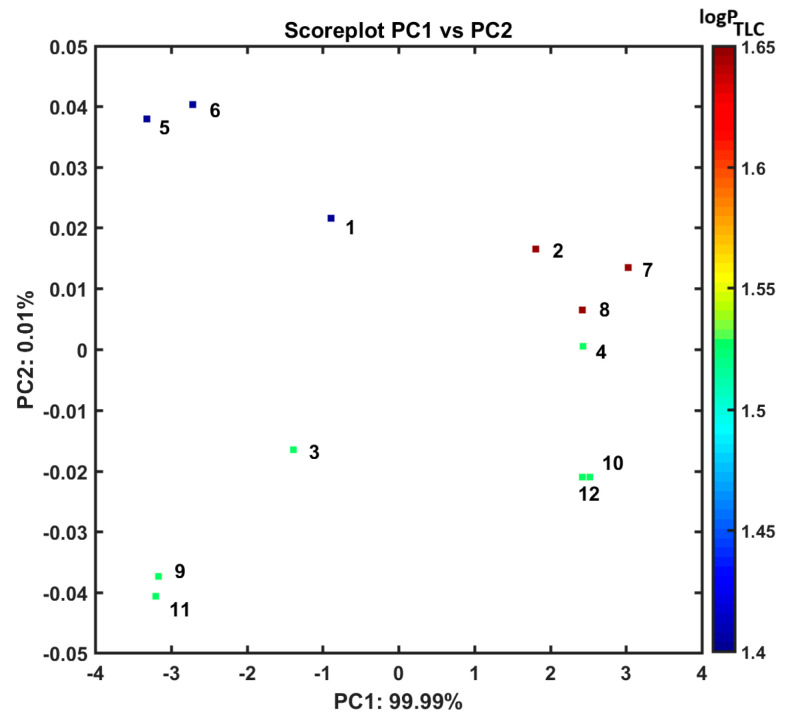
Projection of liposomal systems **1**–**12** on plane defined by PC1 and PC2. Colors code empirical TLC lipophilicity of 9-PBThACl and DOX molecules in logarithmic scale (logP_TLC_), where **1**: [L_DPPCl/DOX_], **2**: [L_DPPC/9-PBThACl_], **3**: [L_DPPC/9-PBThACl/DOX_]_λ_, **4**: [L_DPPC/9-PBThACl/DOX_]_λ2_, **5**: [L_DPPCl/DOX_]:HSA, **6**: [L_DPPCl/DOX_]:dHSA, **7**: [L_DPPC/9-PBThACl_]:HSA, **8**: [L_DPPC/9-PBThACl_]:dHSA, **9**: [L_DPPC/9-PBThACl/DOX_]_λ1_:HSA, **10**: [L_DPPC/9-PBThACl/DOX_]_λ2_:HSA, **11**: [L_DPPC/9-PBThACl/DOX_]_λ1_:dHSA, and **12**: [L_DPPC/9-PBThACl/DOX_]_λ2_:dHSA.

As a matter of fact, the grouping tendency of the analyzed liposomal complexes can be examined via retracing the (dis)similarities in the multidimensional (mD) parameter’s space of drug release potency produced empirically using UV/Vis measurements. In practice, the drug release profiles generated at temperatures of 37 °C and 41 °C and an environmental pH of 7.4 for the liposomal systems composed of 9-PBThACl and DOX molecules with HSA and dHSA were applied as entry descriptors to the principal component analysis (PCA) as well as the hierarchical clustering analysis (HCA). At first, the metric-related (distance-based) drug release assessment was performed on a pool of 37 absorbance measurements organized into matrix X_12 × 37_ with rows representing various **1**–**12** liposomal systems (**1**: [L_DPPCl/DOX_], **2**: [L_DPPC/9-PBThACl_], **3**: [L_DPPC/9-PBThACl/DOX_]_λ1_, **4**: [L_DPPC/9-PBThACl/DOX_]_λ2_, **5**: [L_DPPCl/DOX_]:HSA, **6**: [L_DPPCl/DOX_]:dHSA, **7**: [L_DPPC/9-PBThACl_]:HSA, **8**: [L_DPPC/9-PBThACl_]:dHSA, **9**: [L_DPPC/9-PBThACl/DOX_]_λ1_:HSA, **10**: [L_DPPC/9-PBThACl/DOX_]_λ2_:HSA, **11**: [L_DPPC/9-PBThACl/DOX_]_λ1_:dHSA, and **12**: [L_DPPC/9-PBThACl/DOX_]_λ2_:dHSA, where λ_1_ = 233 nm and λ_2_ = 488 nm) and columns representing numerical parameters (descriptors) of the recorded experimental absorbance. In order to highlight the absorbance variations (differences), the resulting matrix was centered by subtracting the particular column mean values from each absorbance value at the preprocessing step. Roughly speaking, the PCA method is a projection procedure used to model multivariate data with a relatively small number of so-called principal components (PCs) that enables not only the reduction in data dimensionality (mD → 2/3D) but also graphical data presentation and distance-based similarity evaluation. In practice, the input matrix, X, is decomposed into two mutually orthogonal matrixes of scores (T) and loadings (P)—the principal components (PCs) are constructed as linear combinations of original variables to maximize the description of data variance [55]. Ordinarily, a restricted number of the relevant PCs is selected, taking into account the percentage of the modeled data variance. In our case, the first two PCs account for 99.9% of the total data variance; therefore, the investigated liposomal systems were projected on plane PC1 vs. PC2 which was color-coded according to the experimental TLC lipophilicity of 9-PBThACl and DOX molecules, as illustrated in Figure 6.

Noticeably, the examined liposomal conjugates (**1**–**12**) are generally clustered into two main groups according to the first principal component (PC1). Basically, DOX-containing liposomes compose the first group with PC1 < 0, while liposomes with the encapsulated 9-PBThACl molecule are clustered together along the positive values of the PC1 axis (PC1 > 0). Moreover, the thought-provoking distributions of liposome–drug–albumin complexes can be observed along the second principal component (PC2), where double-drug systems such as 9-PBThACl and DOX (named a ‘combo’) are placed on the negative side of the PC2 axis (PC2 < 0) and are discernibly separated from liposomes containing a single drug molecule (9-PBThACl or DOX).

**Figure 7:**
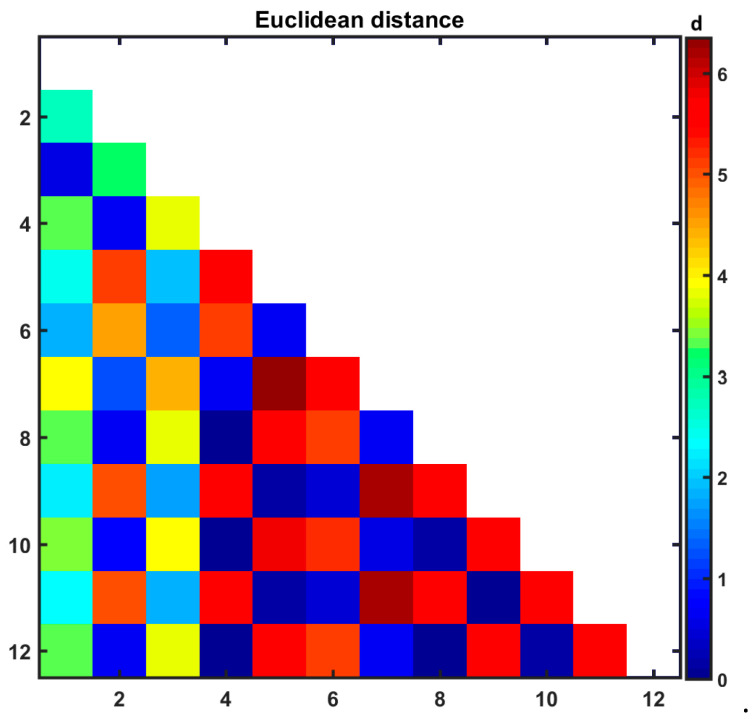
Color-coded matrix of Euclidean distances calculated for liposomal systems **1**–**12**, where **1**: [L_DPPCl/DOX_], **2**: [L_DPPC/9-PBThACl_], **3**: [L_DPPC/9-PBThACl/DOX_]_λ_, **4**: [L_DPPC/9-PBThACl/DOX_]_λ2_, **5**: [L_DPPCl/DOX_]:HSA, **6**: [L_DPPCl/DOX_]:dHSA, **7**: [L_DPPC/9-PBThACl_]:HSA, **8**: [L_DPPC/9-PBThACl_]:dHSA, **9**: [L_DPPC/9-PBThACl/DOX_]_λ1_:HSA, **10**: [L_DPPC/9-PBThACl/DOX_]_λ2_:HSA, **11**: [L_DPPC/9-PBThACl/DOX_]_λ1_:dHSA, and **12**: [L_DPPC/9-PBThACl/DOX_]_λ2_:dHSA.

The Euclidean distances (d) were calculated using PC1 and PC2 coordinates for all **1**–**12** liposomal formulations and introduced as a color-coded triangular matrix in Figure 7. It was confirmed that liposomal complexes **5**, **6**, **11**, and **12** (DOX-based systems) are characterized by the extreme values of PC1 and PC2 coordinates being situated on the edges of the PC1 vs. PC2 plane.

**Figure 8:**
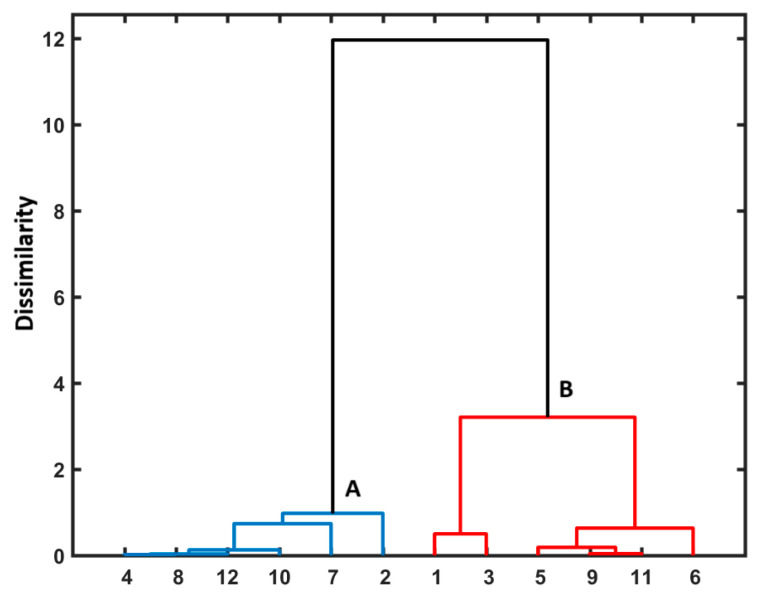
Dendrogram of liposomal conjugates **1**–**12**. A,B are cluster names; **1**: [L_DPPCl/DOX_], **2**: [L_DPPC/9-PBThACl_], **3**: [L_DPPC/9-PBThACl/DOX_]_λ_, **4**: [L_DPPC/9-PBThACl/DOX_]_λ2_, **5**: [L_DPPCl/DOX_]:HSA, **6**: [L_DPPCl/DOX_]:dHSA, **7**: [L_DPPC/9-PBThACl_]:HSA, **8**: [L_DPPC/9-PBThACl_]:dHSA, **9**: [L_DPPC/9-PBThACl/DOX_]_λ1_:HSA, **10**: [L_DPPC/9-PBThACl/DOX_]_λ2_:HSA, **11**: [L_DPPC/9-PBThACl/DOX_]_λ1_:dHSA, and **12**: [L_DPPC/9-PBThACl/DOX_]_λ2_:dHSA.

Briefly, the exploratory HCA procedure generates the clustering pattern of objects/molecules illustrated as a 2D dendrogram produced in the Euclidean-based distance, where the OX axis presents the order of objects (or parameters) and the OY one shows the (dis)similarity between them that is basically related to the clusters’ employed linkage method—Ward’s linkage algorithm is usually employed [56]. In order to confirm the PCA grouping tendency of the investigated liposomal complexes, the HCA method was applied. Similarly to our previous PCA findings (Figure 6), the DOX-containing liposomes form cluster B which varies from collection A with the 9-PBThACl molecule mainly being encapsulated inside liposomes, as presented in the dendrogram in Figure 8.

### 3.4. One-Factor Analysis of Variance (ANOVA)

An analysis of variance (ANOVA) is one of the most commonly used statistical methods in medical research, focusing on the difference in variances between two or more groups of data [57]. In other words, an ANOVA compares the means of at least two data groups to determine whether statistical evidence shows that the means of the associated population vary significantly according to the chosen factor of interest. In the one-factor version of the ANOVA procedure, the null hypothesis (*H*_0_) assumes that all *k* population means (*µ*) are equal, whereas at least one of the *k* population means is not equal to the others according to the alternative hypothesis (*H*_1_). To test the validity of the null hypothesis, one should check whether the contribution of the tested factor is larger with respect to the contribution of the errors (for instance, the random error). If the variances between groups are noticeably large compared to the variances within particular groups of data, then the *H*_0_ hypothesis is not true (it can be rejected) based on the assumed level of significance *p* (typically *p* = 0.05). In practice, the statistical equality of the group’s means is verified by comparing the calculated statistics *F* (so-called *F*-value) with the tabulated critical values of *F*-distribution *F_p(df1, df2)_* on the appropriate degrees of freedom (*df*) and *p* values.

In order to investigate whether there is a statistically significant impact of temperature and the environmental pH on the degree of encapsulated drug release, a classical one-way analysis of variance (ANOVA) was used on the absorption spectra of liposomal systems **1–12**. At first, we assumed in the null hypothesis *H*_0_ that there is no statistical difference between the means of absorbance for liposomal complexes **1–12** in different environmental pH values, where pH_1_ = 5.5, pH_2_ = 6.0, pH_3_ = 6.5, and pH_4_ = 7.4 were recorded in a constant temperature of T = 37 °C. The calculated *F* statistics (see Table S3 in the Supplementary Materials) for all of the analyzed liposomal systems were considerably larger (by at least a few orders of magnitude) with regard to the critical *F*-value (*F*_0.05 (3,144)_ = 3.144) at a significance level of 0.05, indicating that the null hypothesis can be withdrawn and the alternative *H*_1_ stating that there is an important environmental pH effect can be accepted simultaneously. In the next step, the dependences of absorbance measured for all **1–12** liposomal conjugates at a constant pH = 7.4 (physiological liquid) were analyzed in relation to different temperatures, T, where T_1_ = 37 °C and T_2_ = 41 °C, respectively. Similarly to the previous findings (see Table S4 in the Supplementary Materials), almost all calculated *F*-values were noticeably larger in comparison to the critical *F* statistics (*F*_0.05(1,72)_ = 3.97), revealing the statistical importance of temperature on the drug release process. Only in one case, probably due to the systematic error related to the spectrometer calibration process, the absorbance data were accidentally disrupted.

Overall, the influence of the surrounding pH and temperature on the drug release characteristics of liposomal conjugates **1–12** was statistically confirmed, which is in line with our experimental findings as well.

## 4. Conclusions

The provided empirical and statistical findings prove that the practical application of the mREV approach is appropriate for preparing stable liposome-based drug release systems with prospects to be therapeutically implemented. The modified methodology enabled the examination of the impact of temperature and pH on the leakage process of 9-PBThACl and DOX from L_DPPC/9-PBThACl/DOX_ liposomes. Moreover, the analysis of the interaction of the generated liposomes with human serum albumin was described. In order to evaluate the physicochemical properties of the liposomal structure L_DPPC/9-PBThACl/DOX_, the stability of the preparation and the release degree of encapsulated drugs were assessed. The process of encapsulation of the tested compounds in liposomes did not include the impact of HSA/dHSA. Encapsulation was analyzed in terms of the influence of the environmental pH on the final liposomal form of both 9-PBThACl and doxorubicin. The albumin used in the studies provides insights into the physiological conditions and processes that liposomes may undergo. According to data in the literature, albumin molecules partially penetrate the bilayer phospholipid, stimulating the release process of the tested compounds. The fact that both 9-PBThACl and DOX molecules bind with albumin after being released from liposomes is also meaningful. The analysis of the encapsulation degree in liposomes of 9-PBThACl and doxorubicin showed some competition between the studied molecules since relatively high values of encapsulation efficiency (EE%) were only observed for liposomal complexes containing one compound; for example, the _LDPPC/9-PBThACl/DOX_ complex at pH = 7.40 was characterized by encapsulation that was approximately two times lower compared to the L_DPPC/9-PBThACl_ and L_DPPC/DOX_ systems.

The theoretical parameters of liposome-based drug leakage modeling were established based on six selected mathematical models (first-order, Bhaskas, Higuchi, Ritger–Peppas, Korsmeyer–Peppas, and an extension of the classical Freundlich model) implemented to describe the release characteristics of the prospective drug molecule (9-PBThACl) and to compare it with the marketed drug (doxorubicin). In this context, the influence of two temperature points (37 °C and 41 °C) and four pH values, including 5.5, 6.0, 6.50, and 7.4, were investigated in detail. It was revealed that the extension of the classical Freundlich model was characterized by the highest R^2^_adj_ coefficient value. In contrast, the Higuchi model showed the lowest agreement with the empirical data, with R^2^_adj_ values ranging from 0.05669 to 0.99391; however, a complete lack of agreement was recorded as well (R^2^_adj_ ≈ 0).

Not surprisingly, the obtained experimental and modeling data confirm the impact of the surrounding pH on the physicochemical profile of the produced liposomal complexes.

Moreover, the PCA and HCA revealed that the examined liposomal conjugates are generally clustered into two main groups. In general, DOX-containing liposomes compose the first group, while liposomes with the encapsulated 9-PBThACl molecule are clustered together. Finally, the impact of operational conditions (surrounding pH and temperature) on the drug release characteristics of liposomal conjugates was statistically confirmed by the ANOVA method, which is in line with our experimental findings as well.

The presented results include an in-depth characterization of the new liposomal system L_DPPC/9-PBThACl/DOX_, particularly the interactions of HSA molecules with the phospholipid bilayer, thus giving insights into the critical process of drug release from non-specific carriers such as liposomes. It was proven that albumin can directly bind to the surfaces of liposomes, influencing the stability and mechanical properties of their phospholipid bilayers. These interactions can lead to changes in the liposome structure, such as the destabilization of the bilayer and the increased permeability of the liposome membrane to the drug, which facilitates drug release.

## Data Availability

The datasets analyzed during the current study are available from the corresponding author upon reasonable request.

## Supplementary Materials

The following supporting information can be downloaded at: https://www.mdpi.com/article/10.3390/pharmaceutics17020202/s1, Table S1: Fitting parameters of 9-PBThACl/DOX release profiles from [_LDPPC/9-PBThACl/DOX_]:HSA liposomes in different kinetic models; Table S2: Fitting parameters of 9-PBThACl/DOX release profiles from [L_DPPC/9-PBThACl/DOX_]:dHSA liposomes in different kinetic models; Table S3: Statistical parameters of ANOVA for **1**–**12** liposomal complexes in different environment pH values, where pH_1_ = 5.5, pH_2_ = 6.0, pH_3_ = 6.5 and pH_4_ = 7.4 recorded in the constant temperature T = 37 °C. Critical *F*-value at significance level of 0.05 is *F*_0.05(3,144)_ = 3.144; Table S4. Statistical parameters of ANOVA for **1**–**12** liposomal complexes in different temperatures T_1_ = 37 and T_2_ = 41 °C recorded at the constant pH = 7.4 (physiological liquid). Critical *F*-value at significance level of 0.05 is *F*_0.05(1,72)_ = 3.97.
