# Supplementary material for: Preparing a Liposome-Aided Drug Delivery System: The Entrapment and Release Profiles of Doxorubicin and 9-(N-Piperazinyl)-5-methyl-12(H)-quino [3,4-b][1,4]benzothiazinium Chloride with Human Serum Albumin
Source: Pharmaceutics. 2025 Feb 6;17(2):202. doi: 10.3390/pharmaceutics17020202 (PMC11860059; doi:10.3390/pharmaceutics17020202)
Supplement: Supplementary file 1 [file pharmaceutics-17-00202-s001.zip › pharmaceutics-3415168-supplementary.pdf]

Supplementary Materials

Towards Liposome-Aided Drug Delivery System: Entrapment and Release Profile of Doxorubicin and 9-(*N*-piperazinyl)-5-methyl-12(*H*)-quino[3,4-*b*][1,4]benzothiazinium Chloride with Human Serum Albumin

Danuta Pentak [1], Violetta Kozik [2], Andrzej Zieba [3], Marlena Paździor-Heiske [2], Aleksandra Szymczyk [2] , Josef Jampilek [2,*] and Andrzej Bak [2,*]

[1] Faculty of Chemistry and Pharmacy, University of Opole, Oleska 48, 45-052 Opole, Poland
[2] Institute of Chemistry, University of Silesia, Szkolna 9, 40-006 Katowice, Poland
[3] Department of Organic Chemistry, Faculty of Pharmaceutical Sciences in Sosnowiec, Medical University of Silesia in Katowice, Jagiellońska 4, 41-200 Sosnowiec, Poland
* Correspondence: Josef.Jampilek@gmail.com (J.J.); Andrzej.Bak@us.edu.pl (A.B.); Tel.: +48 323591197

Table S1. Fitting parameters of 9-PBThACl/DOX release profiles from $[L_{DPPC/9\text{-}PBThACl/DOX}]$:HSA liposomes in different kinetic models.

**Liposome/pH/Temperature**	**Parameter**	**Mathematical Model**					
		**First-Order**	**Bhaskas**	**Higuchi**	**Ritger-Peppas**	**Korsmeyer-Peppas**	**An extension of classical Freundlich**
$[L_{DPPC/\underline{9\text{-}PBThACl}/DOX}]$:HSA/pH=5.5/37°C	*SUM*	0.06594	0.06861	1.27683	0.58973	0.40403	0.14146
	α	-4.74859	-5.12524×10^{-24}	-66.49279	-1.18795×10^{-55}	-	-110.84737
	k	0.02023	0.02521	0.05708	0.16855	0.24786	110.72547
	n	-	-	-	0.32647	0.25272	0.00183
	R^2_{adj}	0.98476	0.98414	0.70483	0.86226	0.88783	0.96032
$[L_{DPPC/9\text{-}PBThACl/\underline{DOX}}]$:HSA/pH=5.5/37°C	*SUM*	0.03579	0.12254	1.9106	0.984	0.69943	0.45395
	α	0.1576	-5.18709×10-24	-162.83202	-4.67862×10-71	-	-222.96716
	k	0.03928	0.04275	0.05065	0.25608	0.38387	223.09021
	n	-	-	-	0.25615	0.17801	7.3537×10-4
	R^2_{adj}	0.98989	0.96534	0.4595	0.71876	0.74193	0.83076
$[L_{DPPC/\underline{9\text{-}PBThACl}/DOX}]$:HSA/pH=5.5/41°C	*SUM*	0.07663	12.96616	0.09688	0.00275	0.00166	2.02551×10^{-4}
	α	-19.92808	55.19242	-10.55504	-8.73684×10^{-47}	-	-0.05855
	k	0.00874	0.00317	0.05773	0.09575	0.09982	0.12855
	n	-	-	-	0.40966	0.4017	0.36689
	R^2_{adj}	0.98439	-2.1342	0.98026	0.99943	0.99962	0.99995
$[L_{DPPC/9\text{-}PBThACl/\underline{DOX}}]$:HSA/pH=5.5/41°C	*SUM*	0.70582	9.39986	1.73058	0.00198	0.00247	3.71956×10^{-8}
	α	39.05572	174.81655	10	10	-	-0.0197
	k	0.00635	0.00328	0.04365	0.00187	0.00102	0.00141
	n	-	-	-	1.09631	1.19761	1.14477
	R^2_{adj}	0.93999	-0.58574	0.79978	0.99977	0.99971	1
$[L_{DPPC/\underline{9\text{-}PBThACl}/DOX}]$:HSA/pH=6.0/37°C	*SUM*	0.09086	0.02379	0.64167	0.16865	0.11691	0.01376
	α	-16.58021	-1.42518×10^{-21}	-46.03559	-6.48174×10^{-56}	-	-11.07238
	k	0.01277	0.01903	0.05671	0.15578	0.19519	10.9166
	n	-	-	-	0.33169	0.28869	0.01754
	R^2_{adj}	0.97838	0.99434	0.84734	0.95946	0.9671	0.99609
$[L_{DPPC/9\text{-}PBThACl/\underline{DOX}}]$:HSA/pH=6.0/37°C	*SUM*	0.09524	0.01739	0.7762	0.22939	0.16079	0.02294
	α	-14.3182	-1.33136×10^{-22}	-54.34042	-1.28078×10^{-57}	-	-45.65522
	k	0.01417	0.02088	0.05647	0.16683	0.21429	45.50996
	n	-	-	-	0.3214	0.27362	0.00437
	R^2_{adj}	0.97685	0.99577	0.81132	0.94366	0.95328	0.99327

	SUM	100.6343	2.45452	nd[a]	8.95075	2.96959	1.2022×10^{-6}
	α	220.3816	220.3816	nd[a]	337	-	1.01628
[$L_{DPPC/9\text{-}PBThACl/DOX}$]:HSA/pH=6.0/41°C	k	0.00321	0.00321	nd[a]	0.5928	6.56584	-9.41712×10^{-5}
	n	-	-	nd[a]	0	-0.48682	1.59565
	R^2_{adj}	-3.87852	-3.95378	nd[a]	-0.02062	0.66484	1
	SUM	8.04349	4.42356	nd[a]	8.04349	2.84015	1.14552×10^{-4}
	α	-3.66099×10^{9}	146.52659	nd[a]	337	-	1.00751
[$L_{DPPC/9\text{-}PBThACl/DOX}$]:HSA/pH=6.0/41°C	k	1.65888×10^{-10}	0.00297	nd[a]	0.45523	2.32341	-0.00821
	n	-	-	nd[a]	0	-0.34503	0.82662
	R^2_{adj}	-0.0102	-2.65212	nd[a]	-0.02062	0.62945	0.99998
	SUM	0.02187	0.26	0.43319	0.28135	0.21196	0.04777
	α	-5.02695	-5.93272×10^{-22}	-4.32155	-1.79014×10^{-43}	-	-1.82164
[$L_{DPPC/9\text{-}PBThACl/DOX}$]:HSA/pH=6.5/37°C	k	0.01084	0.01239	0.05855	0.0776	0.10603	1.53763
	n	-	-	-	0.44974	0.39181	0.10496
	R^2_{adj}	0.99634	0.95644	0.92742	0.95238	0.96069	0.99105
	SUM	0.03258	14.36994	0.12344	0.09746	0.07273	0.01488
	α	-7.99417	68.5576	-9.79387×10^{-9}	-1.35027×10^{-60}	-	0.43966
[$L_{DPPC/9\text{-}PBThACl/DOX}$]:HSA/pH=6.5/37°C	k	0.00874	0.00278	0.05643	0.06189	0.0763	0.31555
	n	-	-	-	0.48271	0.44398	0.26008
	R^2_{adj}	0.9946	-1.72888	0.97953	98367	0.98681	0.99727
	SUM	77.60973	3.45562	nd[a]	10.37142	4.56237	0.02943
	α	186.748	186.748	nd[a]	350	-	1.04988
[$L_{DPPC/9\text{-}PBThACl/DOX}$]:HSA/pH=6.5/41°C	k	0.00286	0.00286	nd[a]	0.53565	2.44266	-0.00155
	n	-	-	nd[a]	0	-0.31727	1.11907
	R^2_{adj}	-2.76201	-2.83732	nd[a]	-0.02062	0.54603	0.99704
	SUM	7.63859	5.04028	nd[a]	7.63859	2.54227	1.12119×10^{-4}
	α	-3.77597×10^{9}	138.29735	nd[a]	350	-	1.00932
[$L_{DPPC/9\text{-}PBThACl/DOX}$]:HSA /pH=6.5 /41°C	k	1.48245×10^{-10}	0.00286	nd[a]	0.42853	2.34463	-0.01331
	n	-	-	nd[a]	0	-0.35733	0.73932
	R^2_{adj}	-0.0102	-2.52682	nd[a]	-0.02062	0.64857	0.99998
	SUM	0.03289	0.40786	2.06391	1.20868	0.95801	0.48364
	α	3.97697	-1.11088×10^{-22}	-60.64196	-3.13275×10^{-51}	-	-193.74098
[$L_{DPPC/9\text{-}PBThACl/DOX}$]:HSA/pH=7.4/37°C	k	0.02344	0.02371	0.05702	0.15104	0.23527	193.51569
	n	-	-	-	0.34541	0.2623	0.00115
	R^2_{adj}	0.99576	0.92471	0.619	0.77458	0.79471	0.89528
	SUM	0.05114	0.16822	0.17909	0.05829	0.04042	0.00524
[$L_{DPPC/9\text{-}PBThACl/DOX}$]:HSA/pH=7.4/37°C	α	-15.03988	-1.741×10^{-23}	-8.20825	-7.23391×10^{-44}	-	-0.39426

	k	0.00866	0.01092	0.05572	0.08194	0.09696	0.32746
	n	-	-	-	0.43244	0.40117	0.24738
	R^2_{adj}	0.99039	0.96839	0.96635	0.98893	0.91651	0.99889
[$L_{DPPC/9\text{-}PBThACl/DOX}$]:HSA/pH=7.4/41°C	*SUM*	70.58657	3.64262	nd[a]	9.7402	4.0393	0.01333
	α	184.29338	184.29338	nd[a]	360	-	1.04171
	k	0.00278	0.00278	nd[a]	0.51606	2.45437	-0.00246
	n	-	-	nd[a]	0	-0.32449	1.2314
	R^2_{adj}	-2.72359	-2.79725	nd[a]	-0.02062	0.57059	0.99857
[$L_{DPPC/9\text{-}PBThACl/DOX}$]:HSA/pH=7.4/41°C	*SUM*	7.6396	5.05253	nd[a]	7.6396	2.55113	6.92232×10^{-5}
	α	-3.87488×10^{9}	143.1693	nd[a]	360	-	1.00726
	k	1.45119×10^{-10}	0.00278	nd[a]	0.43016	2.36494	-0.01249
	n	-	-	nd[a]	0	-0.35621	0.7461
	R^2_{adj}	-0.0102	-2.53868	nd[a]	-0.02062	0.64749	0.99999

[a] **nd** – non defined

Table S2. Fitting parameters of 9-PBThACl/DOX release profiles from [$L_{DPPC/9\text{-}PBThACl/DOX}$]:dHSA liposomes in different kinetic models.

Liposome/pH/Temperature	Parameter	Mathematical Model					
		First-Order	Bhaskas	Higuchi	Ritger-Peppas	Korsmeyer-Peppas	An extension of classical Freundlich
	SUM	0.09415	0.02501	0.593	0.13754	0.09594	0.01031
	α	-18.03109	-3.17194×10^{-19}	-45.17254	-1.72296×10^{-55}	-	-4.61748
[$L_{DPPC/\underline{9\text{-}PBThACl}/DOX}$]:dHSA/pH=5.5/37°C	*k*	0.01244	0.01887	0.05684	0.15794	0.19352	4.49781
	n	-	-	-	0.32895	0.29013	0.03876
	R^2_{adj}	0.97733	0.99398	0.85721	0.96654	0.97266	0.99703
	SUM	0.08979	0.06462	0.32431	0.03689	0.01977	0.00196
	α	-24.72875	-0.24593	-33.96062	0	-	-0.41928
[$L_{DPPC/9\text{-}PBThACl/\underline{DOX}}$]:dHSA/pH=5.5/37°C	*k*	0.00998	0.01539	0.05631	0.14009	0.15981	0.45445
	n	-	-	-	0.3458	0.31969	0.19719
	R^2_{adj}	0.97856	0.98457	0.92258	0.9911	0.9945	0.99945
	SUM	0.11353	0.0308	0.54355	0.07651	0.05734	0.00485
	α	-23.25363	-0.30451	-54.9635	-2.61167×10^{-63}	-	-2.33865
[$L_{DPPC/\underline{9\text{-}PBThACl}/DOX}$]:dHSA/pH=5.5/41°C	*k*	0.0119	0.01921	0.05562	0.18323	0.20967	2.30496
	n	-	-	-	0.30065	0.2748	0.06439
	R^2_{adj}	0.97011	0.99189	0.8569	0.97965	0.9818	0.99845
	SUM	0.04163	13.97388	0.1093	0.06236	0.04413	0.00843
	α	-10.23483	62.18766	-1.16753	-9.74418×10^{-51}	-	-0.32687
[$L_{DPPC/9\text{-}PBThACl/\underline{DOX}}$]:dHSA/pH=5.5/41°C	*k*	0.00921	0.00303	0.0585	0.0692	0.08255	0.25667
	n	-	-	-	0.46887	0.43564	0.28516
	R^2_{adj}	0.99276	-1.81285	0.981	0.98905	0.99158	0.99837
	SUM	0.05009	13.01538	0.03932	0.05389	0.03855	0.00734
	α	-4.66555	76.43465	-4.14029×10^{-32}	-1.01513×10^{-67}	-	-0.21958
[$L_{DPPC/\underline{9\text{-}PBThACl}/DOX}$]:dHSA/pH=6.0/37 °C	*k*	0.00862	0.00308	0.05697	0.04567	0.05476	0.14573
	n	-	-	-	0.54154	0.50748	0.36931
	R^2adj	0.99225	-1.43318	0.99391	0.99157	0.99357	0.99876
	SUM	0.04235	13.54491	0.07738	0.09227	0.06872	0.0187
	α	-1.31264	79.51086	-7.32878×10^{-58}	-2.95002×10^{-52}	-	-0.27375
[$L_{DPPC/9\text{-}PBThACl/\underline{DOX}}$]:dHSA/pH=6.0/37 °C	*k*	0.00876	0.00303	0.05669	0.0395	0.04963	0.16216
	n	-	-	-	0.56771	0.52506	0.35842
	R^2adj	0.99384	-1.33033	0.98875	0.98644	0.98929	0.99705
	SUM	91.72243	2.49798	nd[a]	9.08401	4.06881	5.78924×10^{-8}
[$L_{DPPC/\underline{9\text{-}PBThACl}/DOX}$]:dHSA/pH=6.0/41°C	*α*	197.00157	197.00157	nd[a]	330	-	1.00007

	k	0.00303	0.00303	nd[a]	0.57259	2.23512	-4.13046×10^{-4}
	n	-	-	nd[a]	0	-0.20828	1.34367
	R^2_{adj}	-3.56034	-3.63146	nd[a]	-0.02062	0.53809	1
	SUM	89.55618	2.65545	nd[a]	8.95757	3.38557	1.29555×10^{-7}
	α	199.29268	199.29268	nd[a]	330	-	1.00886
$[L_{DPPC/9\text{-}PBThACl/\underline{DOX}}]$:dHSA/pH=6.0/41°C	*k*	0.00313	0.00313	nd[a]	0.56693	3.49705	-3.84807×10^{-4}
	n	-	-	nd[a]	0	-0.37835	1.3574
	R^2_{adj}	-3.53685	-3.6046	nd[a]	-0.02062	0.61819	1
	SUM	0.01966	0.27913	0.82252	0.50428	0.38677	0.10067
	α	-2.00929	-2.43294×10^{-25}	-12.79123	-2.29079×10^{-43}	-	-30.35313
$[L_{DPPC/\underline{9\text{-}PBThACl}/DOX}]$:dHSA/pH=6.5/37 °C	*k*	0.01335	0.01475	0.05937	0.09344	0.13543	29.94551
	n	-	-	-	0.42196	0.35288	0.00804
	R^2_{adj}	0.99667	0.9527	0.86061	0.91366	0.92664	0.98071
	SUM	0.00444	0.55118	0.67093	0.66108	0.58909	0.18114
	α	6.36895	4.58324	4.58324	4.58324	-	-7.19019
$[L_{DPPC/9\text{-}PBThACl/\underline{DOX}}]$:dHSA/pH=6.5/37°C	*k*	0.01318	0.01306	0.06194	0.06265	0.08946	6.64825
	n	-	-	-	0.49737	0.42888	0.03677
	R^2_{adj}	0.99942	0.92286	0.9061	0.90652	0.91755	0.97439
	SUM	130.72812	1.74056	nd[a]	10.50365	5.82055	0.03644
	α	249.08865	249.08865	nd[a]	360	-	1.02511
$[L_{DPPC/\underline{9\text{-}PBThACl}/DOX}]$:dHSA/pH=6.5/41°C	*k*	0.00282	0.00282	nd[a]	0.66537	2.39371	-6.1177×10^{-6}
	n	-	-	nd[a]	0	-0.26332	2.05224
	R^2_{adj}	-4.2109	-4.33045	nd[a]	-0.02062	0.4402	0.99646
	SUM	79.43133	2.90601	nd[a]	8.89309	3.74735	5.41203×10^{-8}
	α	200.38635	200.38653	nd[a]	360	-	1.00009
$[L_{DPPC/9\text{-}PBThACl/\underline{DOX}}]$:dHSA/pH=6.5/41°C	*k*	0.00278	0.00278	nd[a]	0.54154	2.32644	-9.45381×10^{-4}
	n	-	-	nd[a]	0	-0.3033	1.18314
	R^2_{adj}	-3.24701	-3.31335	nd[a]	-0.02062	0.56387	1
	SUM	0.07935	13.10399	0.11417	0.0024	0.00145	1.31321×10^{-4}
	α	-22.3069	55.03449	-13.70671	-1.26869×10^{-48}	-	-0.05829
$[L_{DPPC/\underline{9\text{-}PBThACl}/DOX}]$:dHSA/pH=7.4/37°C	*k*	0.00841	0.00303	0.0563	0.10068	0.10464	0.13388
	n	-	-	-	0.39754	0.39023	0.35682
	R^2_{adj}	0.98339	-2.22264	0.9761	0.99949	0.99966	0.99997
	SUM	0.07586	13.219	0.11974	0.00593	0.00341	2.45332×10^{-4}
	α	-21.13112	55.07061	-12.75609	-3.65614×10^{-47}	-	-0.09334
$[L_{DPPC/9\text{-}PBThACl/\underline{DOX}}]$:dHSA/pH=7.4/37°C	*k*	0.00852	0.00303	0.05654	0.09742	0.10372	0.15155
	n	-	-	-	0.4041	0.39223	0.34116

	R^2_{adj}	0.98435	-2.1936	0.97529	0.99876	0.99921	0.99994
	SUM	70.58657	3.23002	nd[a]	11.09457	5.1831	0.06142
	α	184.29338	180.75833	nd[a]	328	-	1.05812
$[L_{DPPC/9-PBThACl/DOX}]$:dHSA/pH=7.4/41°C	k	0.00278	0.00305	nd[a]	0.55329	2.40278	-9.95802×10^{-4}
	n	-	-	nd[a]	0	-0.31096	1.2118
	R^2_{adj}	-2.72359	-2.84427	nd[a]	-0.02062	0.51928	0.99424
	SUM	7.79076	4.72638	nd[a]	7.79076	2.67383	1.82065×10^{-5}
	α	-3.55538×10^{9}	136.7035	nd[a]	328	-	1.00338
$[L_{DPPC/9-PBThACl/DOX}]$:dHSA/pH=7.4/41°C	k	1.64444×10^{-10}	0.00305	nd[a]	0.44275	2.28748	-0.01037
	n	-	-	nd[a]	0	-0.34984	0.7894
	R^2_{adj}	-0.0102	-2.60627	nd[a]	-0.02062	0.63871	1

Table S3. Statistical parameters of ANOVA for **1**-**12** liposomal complexes in different environment pH values, where pH_1=5.5, pH_2=6.0, pH_3=6.5 and pH_4=7.4 recorded in the constant temperature T=37 °C. Critical *F*-value at significance level of 0.05 is $F_{0.05(3,144)}$=3.144.

Liposomal complex	Source of variation	Statistical parameters of ANOVA			
		Sum of squares (*SS*)	Degree of freedom (*df*)	Mean squares (*MS*)	*F*
	Groups	0.6588	3	0.2196	1067.43
**1.** $[L_{DPPC/DOX}]$	*Error*	0.0296	144	2.1×10^{-4}	-
	Total	0.6884	147	-	-
	Groups	0.3249	3	0.1083	5928.4
**2.** $[L_{DPPC/9\text{-}PBThACl}]$	*Error*	0.0018	144	1.8×10^{-5}	-
	Total	0.3267	147	-	-
	Groups	0.0271	3	0.0090	99.06
**3.** $[L_{DPPC/9\text{-}PBThACl/DOX}]_{\lambda1}$	*Error*	0.0131	144	9.1×10^{-5}	-
	Total	0.0402	147	-	-
	Groups	0.0171	3	0.0057	329.9560
**4.** $[L_{DPPC/9\text{-}PBThACl/DOX}]_{\lambda2}$	*Error*	0.0025	144	1.7×10^{-5}	-
	Total	0.0196	147	-	-
	Groups	4.4873	3	1.4958	8724.41
**5.** $[L_{DPPCl/DOX}]$:HSA	*Error*	0.0247	144	1.7×10^{-4}	-
	Total	4.5120	147	-	-
	Groups	0.5210	3	0.1737	1007.53
**6.** $[L_{DPPCl/DOX}]$:dHSA	*Error*	0.0248	144	1.7×10^{-4}	-
	Total	0.5458	147	-	-
	Groups	1.4836	3	0.4945	8627.36
**7.** $[L_{DPPC/9\text{-}PBThACl}]$:HSA	*Error*	0.0083	144	5.7×10^{-5}	-
	Total	1.4918	147	-	-
	Groups	0.6167	3	0.2056	10496.06
**8.** $[L_{DPPC/9\text{-}PBThACl}]$:dHSA	*Error*	0.0028	144	1.9×10^{-5}	-
	Total	0.6195	147	-	-
	Groups	0.2715	3	0.0905	1932.53
**9.** $[L_{DPPC/9\text{-}PBThACl/DOX}]_{\lambda1}$:HSA	*Error*	0.0067	144	4.7×10^{-5}	-
	Total	0.2782	147	-	-

**10.** [$L_{DPPC/9-PBThACl/DOX}$]$_{\lambda 2}$:HSA	*Groups*	0.0811	3	0.0270	2342.21
	Error	0.0017	144	1.2×10^{-5}	-
	Total	0.0827	147	-	-
**11.** [$L_{DPPC/9-PBThACl/DOX}$]$_{\lambda 1}$:dHSA	*Groups*	0.7019	3	0.2340	2665.26
	Error	0.0126	144	8.7×10^{-5}	-
	Total	0.7146	147	-	-
**12.** [$L_{DPPC/9-PBThACl/DOX}$]$_{\lambda 2}$:dHSA	*Groups*	0.1592	3	0.0531	3359.56
	Error	0.0023	144	1.6×10^{-5}	-
	Total	0.1615	147	-	-

λ_1=233 nm and λ_2=488 nm

Table S4. Statistical parameters of ANOVA for **1**-**12** liposomal complexes in different temperatures T_1=37 and T_2=41 °C recorded at the constant pH=7.4 (physiological liquid). Critical *F*-value at significance level of 0.05 is $F_{0.05(1,72)}$=3.97.

Liposomal complex	Source of variation	Statistical parameters of ANOVA			
		**Sum of squares (*SS*)**	**Degree of freedom (*df*)**	**Mean squares (*MS*)**	***F***
	Groups	0.2828	1	0.2828	2840.91
**1.** [$L_{DPPC/DOX}$]	*Error*	0.0072	72	9.9×10^{-5}	-
	Total	0.2899	73	-	-
	Groups	0.1510	1	0.1510	10655.49
**2.** [$L_{DPPC/9\text{-}PBThACl}$]	*Error*	0.0010	72	1.4×10^{-5}	-
	Total	0.1520	73	-	-
	Groups	0.0332	1	0.0332	438.73
**3.** [$L_{DPPC/9\text{-}PBThACl/DOX}$]$_{\lambda1}$	*Error*	0.0054	72	7.5×10^{-5}	-
	Total	0.0386	73	-	-
	Groups	0.0003	1	0.0003	35.85
**4.** [$L_{DPPC/9\text{-}PBThACl/DOX}$]$_{\lambda2}$	*Error*	0.0006	72	8.3×10^{-6}	-
	Total	0.0009	73	-	-
	Groups	0.0463	1	0.0463	698.45
**5.** [$L_{DPPCl/DOX}$]:HSA	*Error*	0.0048	72	6.6×10^{-5}	-
	Total	0.0511	73	-	-
	Groups	1.9080	1	1.9080	16982.41
**6.** [$L_{DPPCl/DOX}$]:dHSA	*Error*	0.0081	72	1.1×10^{-4}	-
	Total	1.9161	73	-	-
	Groups	0.0130	1	0.0130	2239.84
**7.** [$L_{DPPC/9\text{-}PBThACl}$]:HSA	*Error*	0.0004	72	5.8×10^{-6}	-
	Total	0.0135	73	-	-
	Groups	0.0634	1	0.0634	3770.78
**8.** [$L_{DPPC/9\text{-}PBThACl}$]:dHSA	*Error*	0.0012	72	1.7×10^{-5}	-
	Total	0.0646	73	-	-
	Groups	0.0043	1	0.0043	38.22
**9.** [$L_{DPPC/9\text{-}PBThACl/DOX}$]$_{\lambda1}$:HSA	*Error*	0.0081	72	1.3×10^{-4}	-
	Total	0.0125	73	-	-

**10.** [$L_{DPPC/9-PBThACl/DOX}$]$_{\lambda 2}$:HSA	*Groups*	0.0002	1	0.0002	16.39
	Error	0.0009	72	1.3×10^{-5}	-
	Total	0.0012	73	-	-
**11.** [$L_{DPPC/9-PBThACl/DOX}$]$_{\lambda 1}$:dHSA	*Groups*	0.0003	1	0.0003	1.92
	Error	0.0102	72	1.4×10^{-4}	-
	Total	0.0105	73	-	-
**12.** [$L_{DPPC/9-PBThACl/DOX}$]$_{\lambda 2}$:dHSA	*Groups*	0.0001	1	0.00013	6.27
	Error	0.0015	72	2.1×10^{-5}	-
	Total	0.0016	73	-	-

λ_1=233 nm and λ_2=488 nm
